# Normative Values of Retinal Oxygen Saturation in Rhesus Monkeys: The Beijing Intracranial and Intraocular Pressure (iCOP) Study

**DOI:** 10.1371/journal.pone.0150072

**Published:** 2016-03-01

**Authors:** Jing Li, Yiquan Yang, Diya Yang, Xiangxiang Liu, Yunxiao Sun, Shifei Wei, Ningli Wang

**Affiliations:** 1 Beijing Institute of Ophthalmology, Beijing Tongren Eye Center, Beijing Tongren Hospital, Capital Medical University; Beijing Ophthalmology & Visual Sciences Key Laboratory, Beijing, China; 2 Beijing Tongren Eye Center, Beijing Tongren Hospital, Capital Medical University; Beijing Ophthalmology & Visual Sciences Key Laboratory, Beijing, China; Harvard Medical School, UNITED STATES

## Abstract

**Objective:**

To study the normal values of the retinal oxygen saturation in Rhesus monkeys and to evaluated repeatability and reproducibility of retinal oxygen saturation measurements.

**Methods:**

Eighteen adult Rhesus macaque monkeys were included in this experimental study. An Oxymap T1 retinal oximeter (Oxymap, Reykjavik, Iceland) was used to perform oximetry on all subjects. Global arterial (SaO_2_) and venous oxygen saturation (SvO_2_), arteriovenous difference in SO_2_ were measured. In the first examination, each eye was imaged three times. At the following two examinations, each eye was imaged once. All examinations were finished in one month. P values were calculated to evaluate the difference between the measurements during three visits by performing an ANOVA. Intra-visit and inter-visit intraclass correlation coefficient (ICC) was determined.

**Results:**

At baseline, the average SaO_2_ and SvO_2_ were 89.48 ± 2.64% and 54.85 ± 2.18%, respectively. The global A-V difference was 34.63 ± 1.91%. The difference between the three visits was not significant (p>0.05). The highest A-V difference in SO_2_ and lowest saturations were found in the inferotemporal quadrant. Intra-session and inter-visit repeatability were both high. For all oxygen saturation parameters, the ICC values of the intra-session repeatability ranged between 0.92 and 0.96. As found previously, a relatively high ICC value for inter-visit repeatability also was found for all oxygen saturation measurements, ranging between 0.86 and 0.94, with the lowest values in the infero-nasal quadrant.

**Conclusions:**

Our study is the first to describe retinal SO_2_ in healthy Rhesus monkeys. In normal monkey eyes, the reproducibility and repeatability of retinal oximetry oxygen saturation measurements were high in the retinal arterioles and venules. Our results support that Oxymap T1 retinal oximetry is a suitable and reliable technique in monkey studies.

## Introduction

Oxygen is critical to the retina and normal oxygen levels in ocular tissues are vital for healthy eyes [1]. Many ocular diseases, such as age-related macular degeneration (AMD) [2], diabetic retinopathy [3,4], central retinal vein occlusion (CRVO) [5,6], branch retinal vein occlusion (BRVO) [7], and glaucoma [8,9], have shown abnormal oxygen saturation. With the development of modern spectrophometric retinal oximetry techniques, which allow measurement of differences in absorption wavelengths of oxygenated or deoxygenated hemoglobin, oxygen saturation (SO_2_) in retinal blood vessels can be assessed noninvasively [10]. An instrument that has been developed for this purpose is the Oxymap T1 Retinal Oximeter (Oxymap, Reykjavik, Iceland) [11].

Though oxygen saturation of healthy people (of different ethnicities) or with various ocular diseases has been reported many times [2–9,12–17], the normal values of oxygen saturation and its repeatability and reproducibility in primates are still lacking. We carried out this study to assess the normal values of retinal oxygen saturation in Rhesus monkeys, and we evaluated the repeatability and reproducibility of retinal oxygen saturation measurements. We performed this study in recognition of the primary importance of reproducibility of a technique used to detect diseases and, in particular, the reproducibility of measuring oxygen saturation in monkeys.

## Methods

### Subjects

We used 18 adult male Rhesus macaque monkeys (*Macaca mulatta)* in the study. These monkeys had a mean weight of 7.7 kg (range 7.0–8.7 kg) and a mean age of 6.5 years (range 5.5–7.5 years). Examinations of the monkeys were performed in the morning/afternoon to avoid diurnal variations, which means that some monkeys had exams in the morning and subsequently had follow-up exams in the mornings while other monkeys had exams in the afternoon and subsequently had follow-up exams only in the afternoon. All examinations were finished in one month.

### Ethics statement

The study was carried out in accord with the Declaration of Helsinki and the ARVO Statement for the Use of Animals in Ophthalmic and Vision Research and approved by the Institutional Animal Care and Use Committee (IACUC) of Capital Medical University. The Rhesus monkeys involved in this study were all purchased from Beijing Institute of Xieerxin Biology Resource, which was the largest nonhuman primate center in north part of China and specialized in breeding laboratory nonhuman primate. Then, the Rhesus monkeys were housed at the large animal room of Capital Medical University according to the the national standard of China which was named as Laboratory animal institutions—general requirements for quality and competence (GB/T 27416–2014). In details, each monkey was feeded in a separate cage with the size of 1×1×1.2m^3^. The temperature was 16~28°C, the maximum daily tempetature difference was 4°C, relative humidity was 40~70%, the minimum air changes was eight times per hour, air velocity of animal cages was no more than 0.2m/s, ammonia concentration was no more than 14mg/m3, noise was no more than 60dB, the minimum working intensity of illumination was 200 lx (the International System unit of illumination, equal to one lumen per square meter), animal illumination was 100~200 lx, day and night alternating time was 12/12. Feeding regimens were standard animal feeds and various kinds of vegetables and fruits such as bananas and carrot, every two monkeys owns a sharing space to play and relax in different times, in which many toys and swing were provided. No monkey that participated in this study was sacrificed.

### Image acquisition

None of the monkeys had participated in any previous studies or received any interventions. Previous to examination, the monkeys were anesthetized with ketamine HCL (20 mg/kg, intramuscular injection). They also received repeated injections of ketamine (10 mg/kg) during the examination if needed. Topical tropicamide (0.5%) was used for pupil dilation. A rigid plano contact lens was in place on the cornea during imaging. Artificial tears (Refresh Plus, Allergan, Irvine, CA) were used to maintain corneal moisture.

**Fig 1:**
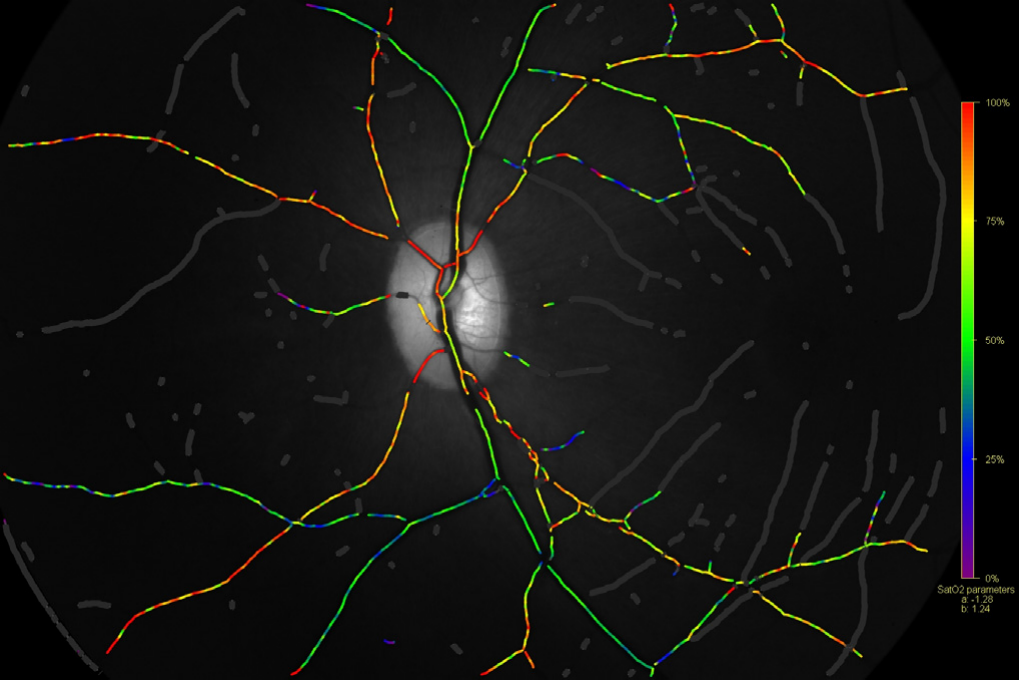
A normal oxymap image with the pseudo-colour saturation overlap map (Background image—570 nm).

Then, an Oxymap T1 Retinal Oximeter was used to obtain retinal oximetry images (50° dual-wavelength [570 nm and 600 nm]; Fig 1). The lowest setting; i.e., 50 W, was used for the aiming light flash intensity. With the TRC 50DX (Topcon, Japan) fundus camera, a large pupil and small aperture were used. Well-trained retinal photographers took all images. A single optic disk-centered fundus image was obtained for each eye. The photographer judged the image quality during image acquisition; images that were not in focus, had bright shadows or reflections, poor positioning or poor contrast were omitted.

### Segment selection and analysis

Version 2.4.2 of the Oxymap Analyzer (Oxymap), which is specialized software, was used to process the retinal oximetry images. A previous report described the Oxymap T1 retinal SO_2_ calculation method in detail. Light absorption spectra differences between deoxyhemoglobin and oxyhemoglobin at 570 nm and 600 nm were determined. The following parameters were measured in our study: SO_2_ in the arterioles (SaO_2_) and venules (SvO_2_), and arteriovenous (A-V) differences in SO_2_.

**Fig 2:**
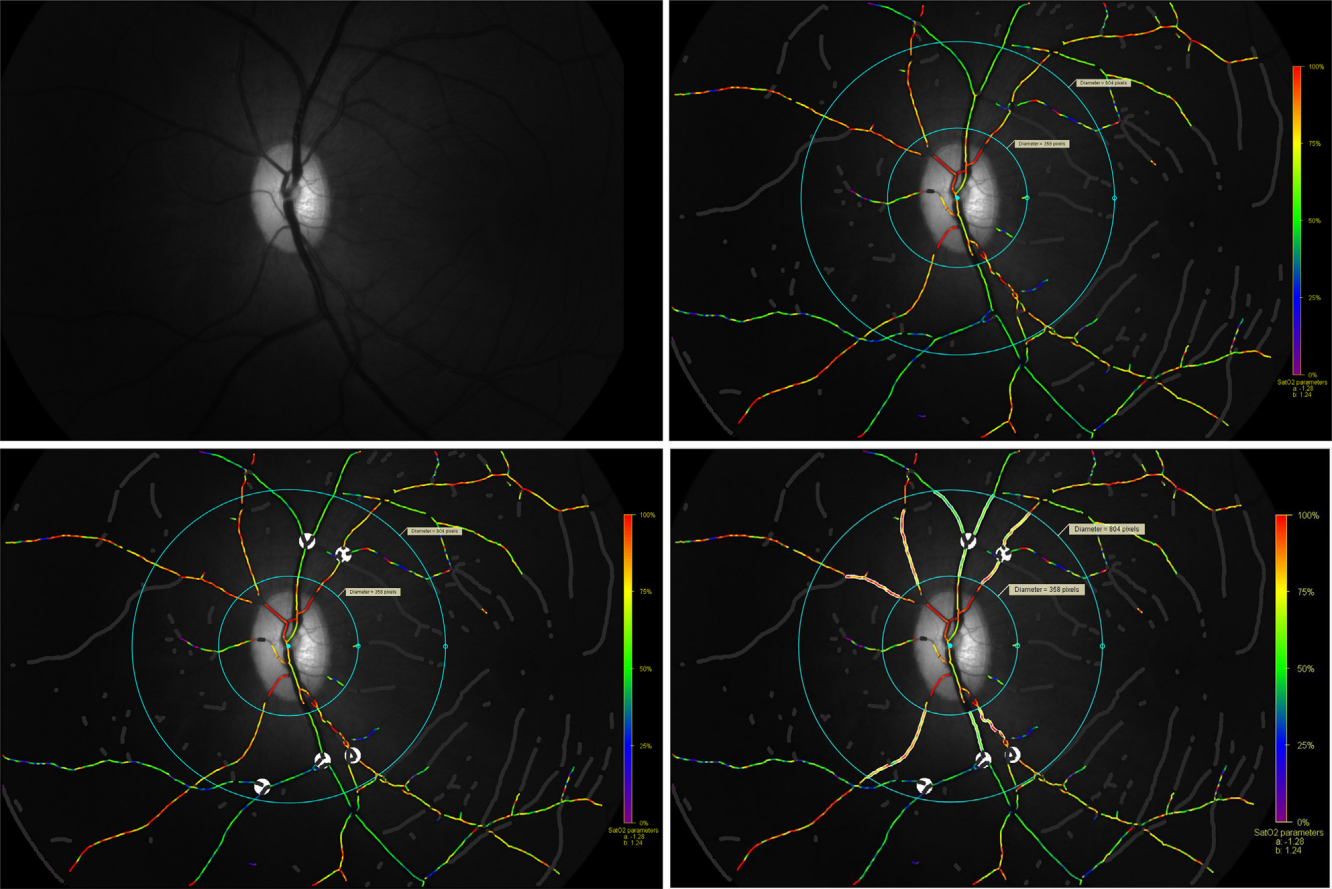
Method of analyzing images. a. 570nm image; b. The vessels were minimum 50 pixels from the optic disc margin and at least 100 pixels from the edge of the image; c. Masking of vessel branchings and A-V crossings along with marking of 2 concentric circles within which vessel segments would be analysed; d. Vessel segment markings (thickest arteriole and venule per quadrant).

We used the protocol described by Geirsdottier et al. [13] to choose sections of the vessels (Fig 2). Arterioles and venules were identified by the following criterion. The vessels that were at least 100 pixels long, at least 8 pixels wide and were a minimum of 50 pixels away from the optic disc margin and at least 100 pixels away from the edge of the image were chosen. Sections were chosen that were after the vessel branchings but were not more than 150 pixels from the optic disc.

### Statistical analysis

Only one eye from each subject was used in the final analysis. Eyes were chosen randomly. SPSS statistics version 17.0 (SPSS, Inc., Chicago, IL, USA) was used for all statistical analyses. We analyzed the retinal vessel oximetry measurement as a continuous variable. The results are presented as mean ± SD. Three retinal oximetry images were obtained during the first examination to assess intra-session repeatability. The intra-session intraclass correlation coefficient (ICC) was calculated. The first retinal oximetry image taken during the initial visit and retinal oximetry image taken in each follow-up visit were collected to determine inter-session reproducibility. The inter-session ICC was also calculated. We also performed an ANOVA and calculated the P value in order to evaluate the difference between the measurements during three visits.

## Results

Eighteen adult male Rhesus macaque monkeys (18 eyes) were used in this study. We studied both eyes of each monkey.

Table 1 shows the quadrant and overall retinal oximetry results and the A-V differences at three times in oximetry values (all P value >0.5). At baseline, the average SaO_2_ and SvO_2_ were 89.48 ± 2.64% and 54.85 ± 2.18%, respectively. The global A-V difference was 34.63 ± 1.91%. The infero-temporal quadrant had the lowest saturations and highest A-V difference in SO_2_.

**Table 1:** Distributions of Retinal Oximetry Levels in Normal Eyes.

Retinal Oximetry	Mean (SD)*	Mean (SD)**	Mean (SD)***	P value
**SaO2, %**				
**Overall**	89.48(4.2)	87.21(3.2)	88.13(4.1)	0.757
**ST**	88.36(3.9)	87.66(3.1)	89.15(2.8)	0.698
**SN**	91.65(3.5)	90.03(4.2)	92.13(5.1)	0.845
**IN**	90.48(4.8)	88.98(3.9)	89.96(3.7)	0.676
**IT**	86.49(5.4)	85.79(4.7)	86.24(3.5)	0.578
**SvO2, %**				
**Overall**	54.85(3.9)	55.54(4.2)	56.14(3.3)	0.919
**ST**	53.97(4.5)	52.37(3.8)	54.23(4.1)	0.897
**SN**	55.75(4.9)	56.88(5.1)	57.25(4.2)	0.869
**IN**	54.92(3.2)	55.18(3.2)	53.18(2.4)	0.746
**IT**	50.39(6.2)	52.15(4.3)	51.46(4.4)	0.828
**A-V difference in SO**_**2**_**, %**				
**Overall**	34.63(3.7)	32.24(3.6)	33.13(3.5)	0.726
**ST**	32.83(5.1)	34.45(3.7)	35.13(3.9)	0.689
**SN**	35.92(5.3)	34.28(4.9)	35.16(2.7)	0.774
**IN**	35.21(4.7)	33.11(4.8)	35.45(5.4)	0.892
**IT**	37.69(3.8)	34.91(4.1)	35.55(3.1)	0.633

*, baseline (exam 1)

**, exam 2

***, exam 3

AV difference, arterio-venous difference; ST, supero-temporal; SN, supero-nasal; IN, infero-nasal; IT, infero-temporal, SaO2, retinal arteriolar oximetry measurement; SvO2, retinal venular oximetry measurement. SD, standard deviation

Table 2 shows the inter-session (inter-visit) and intra-session repeatability for SaO_2_. The range for ICC values of the intra-session repeatability was 0.92 and 0.96 for all oxygen saturation parameters. The lowest values were in the infero-temporal quadrant. There was a relatively high ICC value for inter-session (inter-visit) repeatability for all oxygen saturation results. The range was 0.86 and 0.94 and the lowest values were in the infero-nasal quadrant.

**Table 2:** Intrasession and Intervisit Repeatability of retinal oximerty measurements in 18 Normal Rhesus Monkeys.

Retinal Oximetry Measurements	ICC	95% CI
**Intravisit**		
** SaO2**		
** ST**	0.95	0.87–0.98
** SN**	0.96	0.90–0.98
** IT**	0.94	0.89–0.96
** IN**	0.96	0.82–0.99
** SvO2**		
** ST**	0.93	0.83–0.97
** SN**	0.96	0.93–0.99
** IT**	0.92	0.90–0.95
** IN**	0.94	0.80–0.99
**Intervisit**		
** SaO2**		
** ST**	0.92	0.88–0.97
** SN**	0.91	0.89–0.94
** IT**	0.93	0.83–0.96
** IN**	0.94	0.90–0.99
** SvO2**		
** ST**	0.88	0.81–0.99
** SN**	0.90	0.84–0.98
** IT**	0.89	0.80–0.95
** IN**	0.86	0.82–0.93

AV difference, arterio-venous difference; ST, supero-temporal; SN, supero-nasal; IN, infero-nasal; IT, infero-temporal, SaO2, retinal arteriolar oximetry measurement; SvO2, retinal venular oximetry measurement.

## Discussion

Our study was the first to establish a normative database for retinal SO_2_ in Rhesus monkeys. The Oxymap T1 retinal oximeter was used because it was sensitive to changes in hemoglobin SvO_2_ and SaO_2_. Also, it produced reliable measurements. Every research group must create its own normative database with this technology [12–16].

The distribution results for retinal oximetry levels in normal monkey eyes were the average retinal arteriolar oximetry levels were 89.48% (SD: 4.2%; interquartile range, 88.45%–90.51%) and the average retinal venular oximetry levels were 54.85% (SD: 3.9%; interquartile range, 52.98%–56.72%).

Clinical investigations produced similar results to our study. Yip et al. [14] assessed the reliability and determinants of retinal vessel oximetry measurements using the Oxymap T1 Retinal Oximeter in normal eyes of Asians. They determined that overall and quadrant retinal oximetry measurements, as well as A-V differences had similar oximetry values. The overall retinal venular oximetry value was 54.22% (SD: 6.9%; interquartile range, 52.75%–55.68%) and the arteriolar oximetry result was 93.64% (SD: 6.9%; interquartile range, 92.18%–95.73%). The overall A-V difference value was 39.43% (SD: 8.9%; interquartile range, 37.53%–41.33%). The range for ICC values was 0.85 to 0.96 for intravisit repeatability, which was a little bit lower than our result. Mohan et al. [15] measured retinal arteriolar oximetry levels in normal Asian Indians (98 eyes; mean age 33 years, range 18–63 years; SD: 12.4) and found that they were 90.3% ± 6.6% and retinal venular oximetry levels were 56.9% ± 6.3%.

In experimental settings and clinical routines, it is important to know the repeatability of measurements to determine whether the observed changes are valid or just due to fluctuations of the methods. In our study, results showed that the reproducibility of the measurements for retinal oxygen saturation parameters were similar to the reproducibility found in patients. In our study, the range of ICC values for intra-session repeatability was 0.92 and 0.96 for all oxygen saturation parameters. The lowest values were in the infero-temporal quadrant. Also, there was high ICC value found for inter-session repeatability for all oxygen saturation measurements, ranging between 0.86 and 0.94, with the lowest values in the infero-nasal quadrant. As we all know, different ocular diseases can affect different quadrants. The analysis of oxygenation by quadrant was very important on the research of various ocular diseases. Our study suggested that the repeatability and reproducibility of oxygen saturation retinal oximetry measurements for retinal arterioles and venules in normal monkeys were high, which was similar to reports on human eyes [17–20]. Yip et al. [14] assess the determinants of retinal vessel oximetry measurements and their reliability with an Oxymap T1 Retinal Oximeter in 188 eyes of normal Asian subjects and found that the ICC values ranged from 0.89 to 0.99 for intragrader reliability and 0.85 to 0.96 for intravisit repeatability.

There were some limitations in our study. First, anesthesia may cause a decrease in oxygen saturation; in other words, the actual oxygen saturation of Rhesus macaque monkeys may be higher than the data determined in our study. Second, the results we obtained for Rhesus macaque monkeys may not translate to reproducibility of retinal oximetry in other species. Third, our study employed normal eyes in normal monkeys. These results may not transfer to monkeys with optic nerve damage, such as occurs with glaucoma. Usually, reproducibility has been found to be lower in patients with diseases compared to healthy subjects.

In our study, we provided for the first time retinal SO_2_ measurement results using retinal oximetry in Rhesus Monkeys. There was a high repeatability and reproducibility of retinal oximetry (Oxymap T1 retinal oximeter) assisted measurements for retinal oxygen saturation in normal eyes of normal monkeys. The results from our study indicated that retinal oximetry (Oxymap T1 retinal oximeter) is a suitable and reliable technique in monkey studies.
